# Pneumococcal carriage and serotype distribution among children with and without pneumonia in Mozambique, 2014-2016

**DOI:** 10.1371/journal.pone.0199363

**Published:** 2018-06-26

**Authors:** Tolulope Adebanjo, Fernanda C. Lessa, Helio Mucavele, Benild Moiane, Alberto Chauque, Fabiana Pimenta, Sergio Massora, Maria da Gloria Carvalho, Cynthia G. Whitney, Betuel Sigauque

**Affiliations:** 1 Epidemic Intelligence Service, Centers for Disease Control and Prevention, Atlanta, GA, United States of America; 2 Respiratory Diseases Branch, Division of Bacterial Diseases, National Center for Immunization and Respiratory Diseases, Centers for Disease Control and Prevention, Atlanta, GA, United States of America; 3 Fundação Manhiça, Centro de Investigação em Saúde da Manhiça (CISM), Maputo, Moçambique; 4 John Snow Inc. (JSI) on the Maternal and Child Survival Program–MCSP (USAID Grantee), Maputo, Moçambique; Public Health England, UNITED KINGDOM

## Abstract

**Background:**

Pneumococcal colonization is a precursor to pneumonia, and pneumococcal conjugate vaccines (PCV) can decrease vaccine-type (VT) colonization. Pneumococcal colonization studies are traditionally done among healthy children in the community; however, VT colonization prevalence may differ between these children and those with pneumonia. We assessed overall and VT pneumococcal colonization and factors associated with colonization among children with and without pneumonia after Mozambique introduced 10-valent PCV (PCV10) in 2013.

**Methods:**

We used data from ongoing pneumonia surveillance in children aged <5 years and from cross-sectional nasopharyngeal colonization surveys conducted in October 2014 –April 2015 and October 2015 –May 2016. Pneumonia was defined using WHO standard criteria for radiologically confirmed pneumonia. Children with pneumonia enrolled from January 2014 –April 2016 were compared to children without pneumonia enrolled from the cross-sectional surveys. Clinical data and nasopharyngeal (NP) swabs were collected from each child. NP specimens were cultured for pneumococci, and culture-negative specimens from children with pneumonia underwent polymerase chain reaction (PCR).

**Results:**

Of 778 and 927 children with and without pneumonia, 97.4% and 27.0% were exposed to antibiotics before swab collection, respectively. Based on culture, pneumococcal colonization was 45.1% for children with and 84.5% for children without pneumonia (*P*<0.001); VT pneumococcal colonization was 18.6% for children with and 23.4% for children without pneumonia (*P* = 0.02). The addition of PCR in children with pneumonia increased overall and VT-pneumococcal colonization to 79.2% and 31.1%, respectively. In multivariable analysis including PCR results, pneumonia was associated with VT pneumococcal colonization (adjusted OR: 1.4, 95%CI: 1.10–1.78).

**Conclusion:**

Vaccine-type pneumococcal colonization remains common among children with and without pneumonia post-PCV10 introduction in Mozambique. In a population of children with high antibiotic exposure, the use of PCR for culture-negative NP swabs can improve assessment of pneumococcal colonization and circulating serotypes.

## Introduction

Pneumonia is the single largest infectious cause of death in children worldwide, causing over 900,000 deaths in children under the age of five years [1]. *Streptococcus pneumoniae*, also known as pneumococcus, is the most common cause of bacterial pneumonia in children and is a common colonizer in the nasopharynx [1]. Pneumococcal colonization is highest in young children, especially among those with respiratory symptoms, and colonized children can serve as a reservoir for transmission of pneumococcus to other children and adults [2–4].

Pneumococcal colonization is a necessary precursor to a spectrum of clinical syndromes, which include invasive diseases, such as bacteremia and meningitis, and non-invasive diseases, such as otitis media and pneumonia [2, 5, 6]. The widespread introduction of pneumococcal conjugate vaccine (PCV) has led to reductions in invasive pneumococcal disease and vaccine-type (VT) colonization globally [7–9]. In April 2013, Mozambique added the 10-valent pneumococcal conjugate vaccine (PCV10) to the routine infant immunization program with support from Gavi, the Vaccine Alliance. The vaccine is given as a three-dose primary series, with doses at 2, 3, and 4 months of age and no booster dose; administrative data from 2016 indicates 95% vaccine coverage for all 3 doses in the country [10].

Manhiça District in Mozambique has an ongoing surveillance system for pneumonia. As part of their surveillance activities, nasopharyngeal (NP) swabs are obtained from children admitted to the hospital for pneumonia treatment. Pneumococcal colonization studies are traditionally performed among healthy children in the community; however, in these studies, certain serotypes causing invasive disease, such as serotypes 1 or 5, may be rare, and the prevalence of VT colonization may be lower in healthy children than in patients with respiratory diseases [8, 11–17]. Consequently, the prevalence of VT colonization in healthy children may not reflect the serotypes causing disease.

In order to determine whether colonization with vaccine serotypes, including those not typically found in healthy children, remained high among children with pneumonia after vaccine introduction, we examined pneumococcal colonization in children with and without pneumonia as part of pneumococcal vaccine impact and effectiveness evaluations in Mozambique. Our objectives were to evaluate overall and VT pneumococcal colonization and to explore factors associated with overall and VT colonization in children with and without pneumonia post-PCV10 introduction in Mozambique.

## Materials and methods

### Setting and design

The Centro de Investigação em Saúde de Manhiça (CISM) has been conducting surveillance for pediatric pneumonia hospitalizations since 2014 in Manhiça and Maputo. Surveillance activities are conducted at 1 hospital in Manhiça, Manhiça District hospital (38 pediatric beds in the rural south), and 2 hospitals in Maputo, Maputo Central Hospital (a national referral hospital with 350 pediatric beds in the urban south) and Mavalane General Hospital (66 pediatric beds in the urban south). As part of pneumonia surveillance, NP swabs are collected within 48 hours of admission for children not admitted to the intensive care unit. Chest-X rays are performed on admission for children with clinical signs of pneumonia and interpreted by two independent readers (clinicians). Patients are classified as having radiologically confirmed pneumonia based on the WHO standardized definition [18]. Discordant chest X-ray interpretations are sent to an external radiologist for final determination.

As part of CISM’s pneumococcal vaccine impact and effectiveness evaluations, we utilized data from two cross-sectional pneumococcal nasopharyngeal colonization surveys among children <5 years old from October 2014 through April 2015 and from October 2015 through May 2016 conducted in the cities of Manhiça and Maputo. For both surveys, HIV-infected children presenting for routine HIV care at outpatient clinics in Manhiҫa and Maputo and HIV-uninfected children randomly selected from an existing Demographic Surveillance System (DSS) in Manhiça district were enrolled [19]. Children were considered HIV-infected if HIV DNA polymerase chain reaction (PCR) was positive at any point in life or if HIV rapid test (Uni-Gold^TM^ HIV) was positive in a child aged >18 months [20]. Children were considered HIV-uninfected if HIV PCR or rapid test (for children > 18 months of age) was negative or if they were born from a mother with a documented HIV negative test during pregnancy.

### Participant groups

We defined children without pneumonia as children < 5 years enrolled in either pneumococcal cross-sectional colonization survey. We defined children with pneumonia as children < 5 years hospitalized with radiologically confirmed pneumonia who had a NP swab collected during January 2014-April 2016 as part of the pneumonia surveillance. For both groups, we only included children born after December 10, 2012, who were age-eligible to receive at least one dose of PCV10. We excluded children who received 13-valent PCV.

### Data and sample collection

Prior to enrollment, written consent to participate was obtained from a parent or guardian of each child. Trained staff performed interviews with the parent or guardian of each participant using a standardized questionnaire to collect demographic data, household information, medical history, and vaccination status.

Vaccination status was confirmed from the child’s health card and the health facility registries; a child was considered unvaccinated if the card was missing or blank and the parent or guardian reported no vaccine doses since birth. Vaccination status was considered unknown if the parent or guardian reported that their child had received vaccination beyond doses given at birth, but no written documentation of vaccine receipt was available (no immunization card or no vaccine information in the registry books at the health facilities). We defined a valid PCV10 dose as a dose received at ≥6 weeks of age, ≥21 days from the last dose, and ≥14 days before NP swab collection. HIV status was confirmed through the child’s health card or HIV testing results if available. HIV status was considered unknown in cases where there was no health card or the health card had no HIV information and there was no testing performed.

Nasopharyngeal sample collection followed procedures outlined in the WHO Working Group guidance for detecting upper respiratory carriage of pneumococcus [21]. A sample was collected from each child using calcium alginate swabs. After collection, NP swabs were immediately placed into cryotubes containing 1mL skim milk, tryptone, glucose, glycerol (STGG) transport medium and placed in a cooler with icepacks. Within 4 hours of collection, inoculated STGG was vortexed for 10–20 sec prior to storage in a -70^o^ C freezer.

### Laboratory methods

For NP swab analysis, 200μl of swab-inoculated STGG media was transferred to 5.0 ml Todd Hewitt broth containing 0.5% yeast extract (THY) and 1 ml of rabbit serum and incubated at 35–37°C for six hours. Cultured broth was plated on sheep blood agar and incubated in 5% CO_2_ at 35−37^0^ C. After 18–24 hours of incubation, plates were examined for the appearance of alpha-hemolytic colonies resembling streptococci. Pneumococci were identified by susceptibility to optochin and bile solubility test. Isolates identified as pneumococcus were serotyped by Quellung reaction. Isolates that were not typeable by Quellung were also tested by conventional multiplex PCR for pneumococcal serotyping.

Nearly all children with pneumonia had received antibiotics either as outpatients or in the hospital before the NP swabs were collected; therefore, culture-negative NP swabs from pneumonia patients also underwent real-time PCR targeting *lytA* for detection of pneumococcal DNA [22]. To obtain DNA extracts for PCR reactions, 400μL of the STGG NP-swab were transferred into 1.5 ml cryotubes containing 300 μL of buffer#4 (Roche isolation kit III) and transferred to MagNA Pure Compact instrument using the external lysis protocol according to manufacture instructions. DNA extracts were eluted to 100 μL and stored at -20°C until PCR testing was performed using Quanta Biosciences PerfeCTa® qPCR ToughMix®, Low ROX™, for the *lyt*A assay. For *lytA* positive specimens, multiplex real-time PCR assay for serotype identification was performed using PerfeCTa Multiplex qPCR ToughMix®, Low ROX™ for 21 assays encompassing 37 serotypes (including pneumococcal conjugate vaccine-type serotypes) [23].

### Data management

Data collected from the questionnaires were sent to the CISM Data Center for double entry into a REDCap database. The original questionnaires were stored locally at CISM and consulted for data cleaning; only study investigators had access to these data. Pictures of vaccine cards were taken and stored in the database to avoid errors in transcription of vaccination histories.

### Statistical analysis

For culture only analyses, we defined pneumococcal colonization as isolation of pneumococci by culture on a nasopharyngeal specimen. For culture and PCR analyses, we defined pneumococcal colonization as detection of pneumococci by either culture or PCR on a nasopharyngeal specimen. Because PCR cannot differentiate serotypes within certain serotype groups, we defined VT as serotypes in PCV10 (1, 4, 5, 6B, 7F, 9V, 14, 18C, 19F, 23F) in addition to those serotypes that PCR was not able to distinguish (6A/6B, 7F/7A, 9V/9A, 18C/18F/18B/18A). We also conducted a sensitivity analysis in which we defined VT as only those serotypes in PCV10 identified by culture (1, 4, 5, 6B, 7F, 9V, 14, 18C, 19F, 23F) or PCV10 serotypes that could be distinguished by PCR (1, 4, 5, 14, 19F, 23F). Non-vaccine type (NVT) was defined as: serotypes not included in our VT pneumococcal colonization definition, isolates that were non-typeable by Quellung reaction, and specimens negative for the 37 serotypes tested by multiplex real-time PCR. When multiple pneumococcal serotypes were identified, the child was classified as having VT colonization if at least one serotype fit the VT definition. Because enrollment for children without pneumonia was not ongoing and occurred during two discrete time periods, in contrast to year-round enrollment of children with pneumonia, we created a time variable to adjust for possible differences in overall and VT pneumococcal colonization linked to time period: enrollment before May 1^st^, 2015 (during the first colonization survey) and enrollment on or after May 1^st^, 2015 (during the second colonization survey).

Descriptive analyses of study group characteristics, overall and VT pneumococcal prevalence, and serotype distribution were performed. Differences in baseline characteristics and pneumococcal prevalence between children with and without pneumonia were compared using chi-square for categorical variables and non-parametric tests for continuous variables. Statistical models were built based on the outcomes of interest: overall pneumococcal colonization and VT pneumococcal colonization.

Univariate analyses of the association between individual variables and the outcomes of interest were performed using logistic regression. Separate multivariable models were constructed for each outcome using automated stepwise logistic regression. Candidate variables for each multivariable model were selected through the creation of a directed acyclic graph [24]. Enrollment time was also included as a candidate variable in each model. Pneumonia was retained as the main exposure of interest for each model to evaluate its association with overall and VT pneumococcal colonization. All analyses were performed using SAS software (version 9.4). A 2-tailed *P*-value <0.05 was considered statistically significant.

The colonization surveys were approved by the institutional review boards of the Mozambican Ministry of Health (Ref: 327/CNBS/2014) and of the Centers for Disease Control and Prevention (Protocol # 6321). The ethics committee of the Mozambican Ministry of Health approved the protocol for the pneumonia surveillance (Ref:333/CNBS/13). The Centers for Disease Control and Prevention determined the pneumonia surveillance protocol to be public health non-research.

## Results

### Participant characteristics

We initially enrolled 812 children with and 935 children without pneumonia. A total of 778 eligible children with and 927 eligible children without pneumonia were included in the final analysis. We excluded 34 children with pneumonia because they received PCV13 rather than PCV10 (n = 9), they were already enrolled as a participant (n = 9), or they had no culture or PCR results (n = 16), and 222 children without pneumonia because they received PCV13 (n = 5) or they did not have culture results (n = 3). Six (0.8%) children with pneumonia had a positive blood culture for pneumococcus. Children with pneumonia were younger and ranged in age from 2–38 months, while children without pneumonia ranged in age from 7 weeks– 39 months. Fewer children with pneumonia were enrolled after May 2015 compared to those without pneumonia (42.5% vs 60.2%, *P*<0.0001). Among children with pneumonia, 16.8% were enrolled from a rural site (Manhiça) and 83.2% were enrolled from an urban site (Maputo or Mavalane), while 76.4% and 23.6% of children without pneumonia were enrolled from a rural (Manhiça) or urban area (Maputo), respectively. Children with pneumonia were also less likely to be HIV positive compared to those without pneumonia addition (12.3% vs. 39.5%, *P* < .0001). Vaccination coverage with at least one dose of PCV10 was high in both children with and without pneumonia, at 92.1% and 89.9%, respectively. Further characteristics of the two groups are described in Table 1.

**Table 1:** Characteristics of children aged <5 years with and without pneumonia in Manhiça and Maputo, Mozambique, 2014–2016.

	Pneumonia	Without Pneumonia	*P-*value
	*n*(%)	*n*(%)	
	N = 778	N = 927	
**Enrolled during or after May 2015**	331 (42.5)	558 (60.2)	<0.0001
**Male**	403 (51.8)	478 (51.6)	0.92
**Median age (IQR**^**a**^**), months**	11.9 (7.0–17.8)	14.8 (11.0–21.2)	<0.0001
Age groups			
<12 months	395 (50.8)	283 (30.5)	
12–23 months	307 (39.5)	483 (52.1)	
≥ 24 months	76 (9.8)	161 (17.4)	
**Number of PCV10 Doses**^b^			
3 doses	572 (73.5)	705 (79.5)	<0.0001
1 or 2 doses	144 (18.6)	92 (10.4)	
0 doses	61 (7.9)	90 (10.2)	
*Unknown*	*2 (0*.*3)*	*40 (4*.*3)*	
**HIV status**^**b**^			
HIV positive	72 (12.3)	357 (39.5)	<0.0001
HIV negative	513 (87.7)	547 (60.5)	
*Unknown*	*193 (24*.*8)*	*23 (2*.*5)*	
**Nutritional status**^b^			
No Malnutrition	575 (73.9)	779 (84.3)	<0.0001
Malnutrition^c^	203 (26.1)	145 (15.7)	
*Missing*	*0 (0*.*0)*	*3 (0*.*3)*	
**Site**			
Rural	131 (16.8)	708 (76.4)	<0.0001
Urban	647 (83.2)	219 (23.6)	
**Antibiotic use in the 3 weeks prior to swab collection** ^**d**^	758 (97.4)	250 (27.0)	<0.0001
**Attend daycare**	25 (3.2)	8 (0.9)	0.0005
**Cigarette smoke exposure**	165 (21.2)	144 (15.5)	0.002
**Exclusive breastfeeding**^**b**^^,^^**e**^	714 (91.9)	877 (95.1)	0.007
*Missing*	*1 (0*.*1)*	*5 (0*.*5)*	
**Socioeconomic status**^**b**^^,^^**f**^			
Low	375 (48.2)	631 (68.4)	<0.0001
High	403 (51.8)	292 (31.6)	
*Missing*	*0 (0*.*0)*	*4 (0*.*4)*	
**Median number of people in home (IQR)**^**b**^	6 (5–8)	6 (4–8)	0.14
*Missing*	*5 (0*.*6)*	*0 (0*.*0)*	
**Positive blood culture**	6 (0.8)	—	
**Died**	9 (1.1)	—	

^a^ Interquartile range

^b^ Denominator excludes missing data

^**c**^ weight-for-age Z-score ≤ -2

^**d**^ Includes outpatient or inpatient antibiotic

^**e**^ <6 months and currently breastfeeding or breastfeeding for at least 6 months)

^**f**^ based on maternal education status, low = none or elementary; high = secondary or university

Any antibiotic exposure (including outpatient or in-hospital) in the three weeks prior to swab collection was higher in children with pneumonia compared to children without pneumonia (97.4% vs 27%, *P*<0.0001). Among children with pneumonia, 82.5% (642/778) received antibiotics prior to hospital admission. In addition, almost all children with pneumonia (90.9%) had their swab collected after receiving intravenous antibiotics on hospital admission; most swabs were collected within 48 hours of receiving in-hospital IV antibiotics (83.7%). For children with pneumonia, antibiotic exposure prior to sample collection was also higher in urban areas compared to rural areas (83.9% vs. 16.1%; *P*<0.0007).

### Pneumococcal colonization

Based on culture results alone, the prevalence of pneumococcal colonization was 45.1% (351/778) for children with pneumonia compared to 84.5% (783/927) for children without pneumonia (*P*<0.001); the prevalence of VT- pneumococcal colonization was 18.6% (145/778) for children with and 23.4% (217/927) for children without pneumonia (*P* = 0.02). Previous antibiotic use in the inpatient setting for children with pneumonia significantly affected the ability to culture pneumococci; the prevalence of pneumococcal colonization was 43.3% (306/707) in children who received in-hospital antibiotics prior to swab collection compared to 63.4% (45/71) in children who did not receive in-hospital antibiotics prior to swab collection (*P* = 0.001). Pneumococcal colonization prevalence was 82.4% (206/250) in children without pneumonia with a history of outpatient antibiotic exposure in the three weeks prior to swab collection, which was similar to 85.2% (577/677) prevalence in children without pneumonia with no history of outpatient antibiotic exposure in the three weeks prior to swab collection (*P* = 0.29).

**Fig 1:**
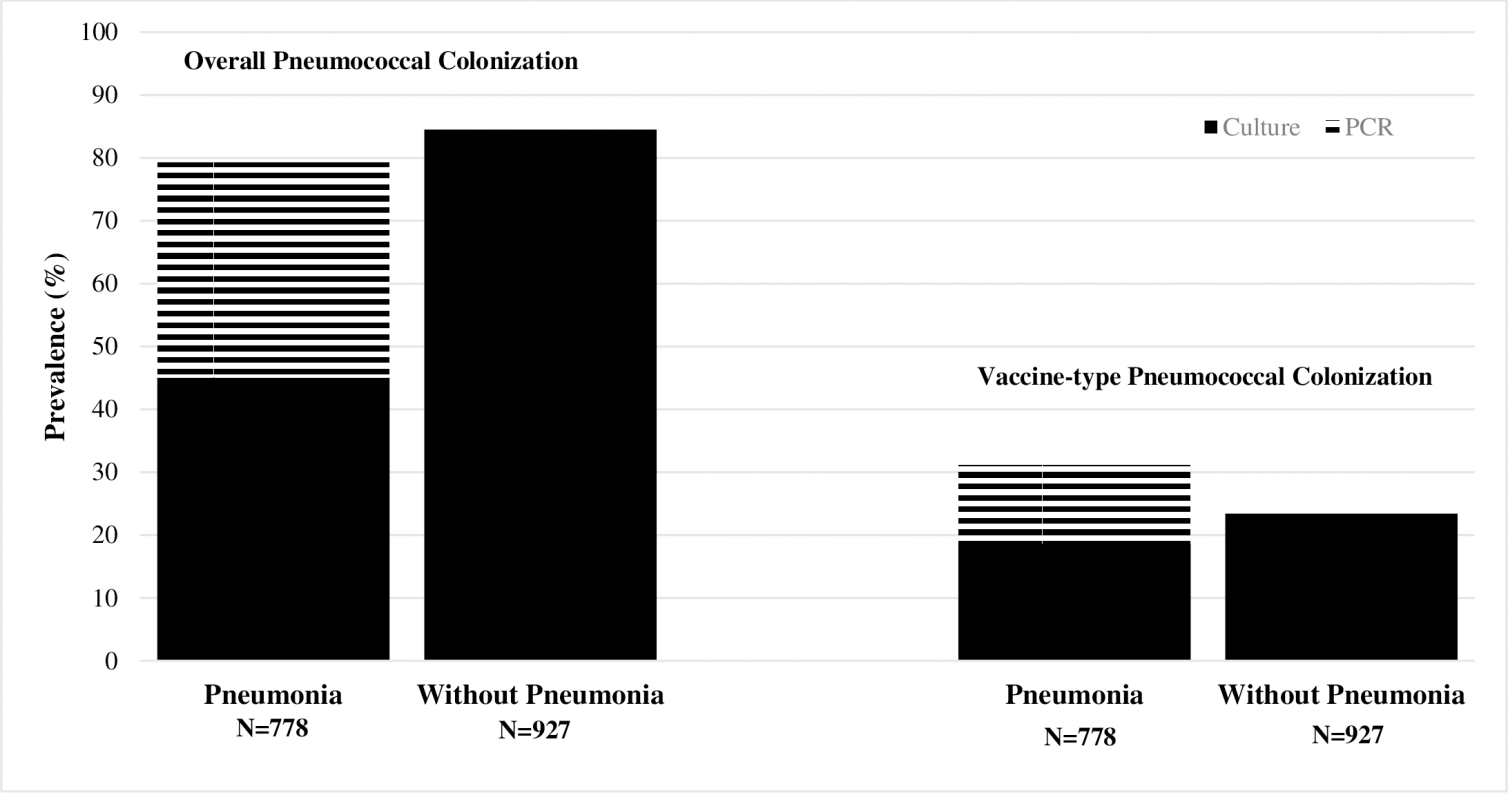
Prevalence of overall and vaccine-type pneumococcal colonization by culture and PCR in children aged <5 years with and without pneumonia in Manhiça and Maputo, Mozambique, 2014–2016.

With the addition of PCR testing of specimens from children with pneumonia, detection of overall and VT pneumococcal colonization increased to 79.2% (616/778) and 31.1% (242/778), respectively. Prevalence of overall pneumococcal colonization remained significantly higher among children without pneumonia compared to those with pneumonia (*P* = 0.005). However, prevalence of VT pneumococcal colonization became significantly higher among children with versus without pneumonia (*P* = 0.0004). (Fig 1).

### Serotype distribution

**Fig 2:**
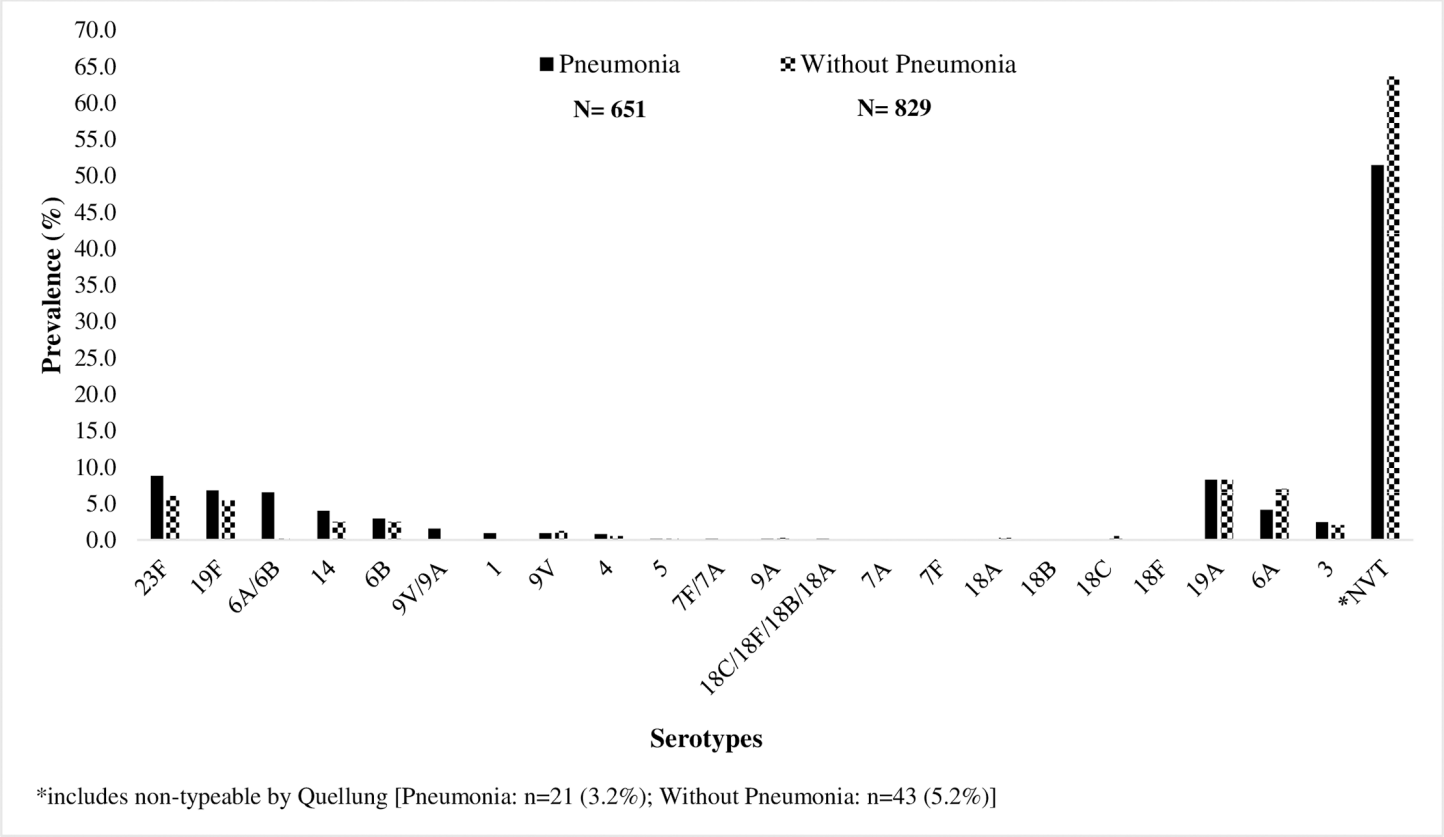
Distribution of serotypes among children aged < 5 years with pneumococcal colonization in Manhiça and Maputo, Mozambique, 2014–2016.

There were a total of 829 serotyping results for children without pneumonia based on culture only and 651 for children with pneumonia using culture or PCR (Fig 2). Among children with pneumonia, the most frequently detected VT serotypes were 6A/6B (13.5%), 23F (8.8%), and 19F (6.8%). Among children without pneumonia, 6A/6B (9.5%), 23F (6.0%), and 19F (5.4%) were also the most common VT serotypes. Serotype 1 accounted for 0.9% (N = 6) of the serotypes found in children with pneumonia (N = 651), but it was not detected in any children without pneumonia. NVTs represented 51.5% of serotypes identified in children with pneumonia and 63.6% of serotypes in children without pneumonia. Among those with pneumococcal colonization detected by culture or PCR, more than one serotype was identified in 5.6% (44/783) of children with pneumonia and 5.4% (33/616) of children without pneumonia (*P* = 0.8).

### Factors associated with overall and vaccine-type pneumococcal colonization

On univariate analysis including PCR results, exclusive breastfeeding (OR: 1.7, 95% CI 1.06–2.60) and rural site (OR: 2.4, 95% CI 1.84–3.11) were associated with overall pneumococcal colonization, while having pneumonia was associated with lower odds of pneumococcal colonization (OR: 0.7, 95% CI 0.55–0.90; Table 2). On multivariable analysis, only living in a rural area remained associated with pneumococcal colonization (OR: 2.5, 95% CI 1.79–3.53). After adjusting for enrollment site, the odds of pneumococcal colonization among children with versus without pneumonia increased to 1.2 (95% CI 0.86–1.66); however, this association was not statistically significant.

**Table 2:** Factors associated with overall pneumococcal colonization in children aged < 5 years in Manhiça and Maputo, Mozambique, 2014–2016.

Variable	Unadjusted OR (95% CI)	Adjusted OR^a^ (95% CI)
**Pneumonia**	0.7 (0.55–0.90)	1.2 (0.86–1.66)
**Age in months**	1.0 (0.99–1.02)	—
**Antibiotic Exposure**	0.7 (0.52–0.88)	—
**Enrollment during or after May 2015**	1.2 (0.97–1.59)	—
**Exclusive Breastfeeding**	1.7 (1.06–2.60)	—
**Daycare**	0.5 (0.23–1.05)	—
**HIV Positive**	0.7 (0.56–0.97)	—
**Number of people in home**	1.0 (0.98–1.05)	—
**Rural site**	2.4 (1.84–3.11)	2.5 (1.79–3.53)

^**a**^ Candidate variables for the model included pneumonia, age, antibiotic exposure, exclusive breastfeeding, number of people in the home, site, daycare enrollment, HIV status, time period enrolled. Pneumonia was required to stay in the model.

Factors associated with VT colonization on univariate analysis included pneumonia (OR: 1.5, 95% CI 1.19–1.83), antibiotic exposure prior to swab collection (OR: 1.3, 95% CI 1.04–1.61), and daycare attendance (OR: 3.4, 95% CI 1.67–6.70; Table 3). Children enrolled on or after May 1, 2015 (OR: 0.7, 95% CI 0.56–0.86) and those from a rural site (OR: 0.8, 95% CI 0.61–0.93) were less likely to be colonized with a VT; both findings were statistically significant. On multivariable analysis including PCR results, pneumonia (OR: 1.4, 95% CI 1.10–1.78) remained associated with VT pneumococcal colonization after controlling for daycare attendance (OR: 2.5, 95% CI 1.04–6.04) and enrollment on or after May 1, 2015 (OR: 0.7, 95% CI 0.52–0.85).

**Table 3:** Factors associated with vaccine-type^a^ pneumococcal colonization in children aged < 5 years in Manhiça and Maputo, Mozambique, 2014–2016.

Variable	Unadjusted OR (95% CI)	Adjusted OR^b^ (95% CI)
**Pneumonia**	1.5 (1.19–1.83)	1.4 (1.10–1.78)
**Age in months**	1.0 (0.97–1.00)	—
**Antibiotic Exposure**	1.3 (1.04–1.61)	—
**Enrollment during or after May 2015**	0.7 (0.56–0.86)	0.7 (0.52–0.85)
**Exclusive Breastfeeding**	0.7 (0.48–1.09)	—
**Daycare**	3.4 (1.67–6.70)	2.5 (1.04–6.04)
**HIV Positive**	0.9 (0.72–1.20)	—
**Number of people in home**	1.0 (0.98–1.04)	—
**Rural site**	0.8 (0.61–0.93)	—
**Number of PCV10 doses**	1.0 (0.88–1.10)	—

^**a**^ Serotypes in the PCV10 vaccine including those that PCR is not able to distinguish

^b^ Candidate variables for the model included age, antibiotic exposure, exclusive breastfeeding, site, number of people in home daycare attendance, HIV status, vaccination status, time period enrolled. Pneumonia was required to stay in the model.

#### Sensitivity analysis

Using a VT definition that included only PCV10 serotypes identified by culture (1, 4, 5, 6B, 7F, 9V, 14, 18C, 19F, 23F) or PCV10 serotypes types distinguished by PCR (1, 4, 5, 14, 19F, 23F), VT prevalence among children with and without pneumonia decreased from 31.1% (242/778) to 20.7% (161/778) and from 23.4% (217/927) to 16.6% (154/927), respectively. The difference in VT carriage prevalence between children with and without pneumonia was still statistically significant (20.7% vs 16.6%, P = 0.03). On multivariable analysis, children with pneumonia had greater odds of VT colonization; however, this was not statistically significant (OR: 1.2, 95% CI 0.93–1.61).

## Discussion

We found that VT-pneumococcal colonization continues to be common in children hospitalized with pneumonia and in children in the community without pneumonia post-PCV10 introduction. The most common colonization serotypes were also similar between the two groups. Serotype 1 was only detected in children with pneumonia, consistent with previous work noting that serotype 1 is rarely found in children with asymptomatic nasopharyngeal colonization [13]. Even though our VT definition was broad to account for the inability of PCR to distinguish 6B, 7F, 9V, and 18C from related serotypes not included in PCV10, children with pneumonia still had a high prevalence of VT colonization after restricting our VT definition for the sensitivity analysis.

When we included results from PCR testing, we observed that VT pneumococcal colonization was about one-third higher in children with pneumonia than in children without pneumonia. Although previous studies have evaluated differences in VT colonization between children with and without pneumonia, findings vary between studies [25–27]. One study performed in Switzerland post-PCV7 implementation found that VT colonization was 41% higher in children with radiographically-confirmed pneumonia compared to healthy children [25]. In this Swiss study, molecular detection of colonization was used in both healthy children and children with pneumonia, and in-hospital antibiotic use prior to swab collection occurred in only approximately 16% of children with pneumonia. In contrast, studies performed pre-PCV7 implementation found that VT colonization was similar between children with and without pneumonia in Israel (29.9% vs 30.9%), and higher among children without versus with pneumonia in Morocco (20.5% vs.16.1%)[26, 27]. Antibiotic use prior to swab collection in children with pneumonia occurred in both of these pre-PCV7 studies; however, only culture was used for pneumococci detection, possibly missing pneumococcal colonization in some children. Unlike these two studies, we used molecular detection to account for antibiotic use prior to sample collection in children with pneumonia, and we evaluated colonization post-vaccine implementation. Our results suggest that future colonization studies might benefit from enrollment of both healthy children and children with clinical syndromes associated with pneumococcal disease, in order to accurately assess prevalence of VT and other circulating serotypes in the community, including those not usually found in healthy children [8, 11, 12, 14, 16, 17].

Almost all (97.4%) of the children with pneumonia in our study were exposed to antibiotics prior to swab collection, compared to only 27.0% of the children without pneumonia; in addition, the type of antibiotic exposure differed between children with and without pneumonia. Children with pneumonia were frequently exposed to intravenous antibiotics in the preceding 24–48 hours before swab collection, and we found that intravenous antibiotics significantly affected our yield in isolating pneumococcus from NP swabs. In contrast, although almost one-fourth of the children without pneumonia had a history of antibiotic exposure in the three weeks preceding swab collection, these children were typically exposed only to oral antibiotics, and the exact timeframe between the last antibiotic dose and swab collection was unknown. As a result, the yield of isolating pneumococcus from NP swabs remained high in children without pneumonia, despite a history of antibiotic exposure. Culture remains the gold standard for detection of pneumococcus in NP swab samples; however, the use of molecular methods that target the *lytA* gene are valuable because antibiotic therapy reduces the yield from culture, while diagnosis by molecular methods can be made after as many as 4 days of antibiotic exposure [22, 28, 29]. Our use of molecular detection resulted in a 75% increase in identification of pneumococcus among children with pneumonia, suggesting that such techniques are a useful tool when evaluating pneumococcal nasopharyngeal colonization in children with pneumonia. However, caution should be taken when using molecular detection on other upper respiratory specimens, as cross-reactivity with other non-pneumococcal streptococci has been reported [30].

The VT colonization prevalence of 31.1% among children with and 23.4% among children without pneumonia that we observed 3 years following PCV10 introduction in Mozambique is quite high compared to other colonization studies. In Kenya, VT colonization prevalence in children from the community was 13% in a colonization survey performed one year after PCV introduction [31]. In both Mozambique and Kenya, prevalence of VT colonization was similar to each other before PCV10 introduction, 33% and 34%, respectively [31, 32]. In developed countries, VT colonization prevalence as low as 3% has been demonstrated among children within three years of PCV13 introduction [33–35]. A vaccine schedule of three primary doses of vaccine without a booster has been shown to reduce VT carriage compared to no PCV in a variety of settings; however, the relative impact on VT carriage of one vaccine schedule over another is unclear because there are few studies in which direct comparisons are made [9]. Our inclusion of children with HIV does not explain the high prevalence of VT carriage, even though such children are at increased risk for pneumococcal disease, as we observed similar VT carriage prevalence in HIV positive (24.9%) and HIV negative (26.4%) children. One possible explanation for the higher proportion of VT serotypes found in our study is that Mozambique did not include catch-up vaccination for older children at the time PCV10 was introduced for young infants, in contrast to the catch-up programs used in Kenya and many other countries from which colonization data are available. More time may be needed to observe the full benefits of PCV programs in settings like Mozambique. Further investigation of vaccine schedule and other host or environmental factors is warranted to explore the high prevalence of VT colonization that remains in Mozambique.

Our study has some limitations. Despite attempts to enroll children with pneumonia from rural sites, most children were enrolled from urban areas where antibiotic exposure was more common. For this reason, we added molecular detection in addition to culture only for pneumonia patients. Although the different methodology for detection of colonization could lead to bias and make direct comparisons between the groups challenging, some studies evaluating the addition of PCR suggest no significant differences in the predominant serotypes identified by culture versus PCR [36–38]. It is also unlikely that the addition of PCR in children without pneumonia would have significantly increased the identification of pneumococcus and VT serotypes because pneumococcal colonization in children without pneumonia was similar to the known pre-vaccine prevalence in the country [39], and PCR increased the prevalence of pneumococcal colonization in children with pneumonia closer to that of children without pneumonia. Further, antibiotic exposure was significantly lower in children without pneumonia and pneumococcal prevalence was similar regardless of antibiotic exposure prior to swab collection, suggesting that antibiotics did not affect pneumococcal colonization in this group. Last, even with the addition of PCR in children with pneumonia, we may have underestimated colonization in this group due to high inpatient antibiotic use prior to swab collection. Another limitation was the difference in HIV prevalence between the two study groups given that the cross-sectional survey in children without pneumonia was designed to enroll a large number of HIV-infected children presenting for routine HIV care. Even though over one-third of the children without pneumonia were HIV-infected, they were generally healthy and on anti-retroviral therapy. Furthermore, in Mozambique, children with HIV infection have previously been shown to have pneumococcal colonization rates similar to healthy children [39], and we observed similar VT colonization between those with and without HIV infection in our study. HIV status was missing for 25% of children with pneumonia due to unavailability of testing supplies; however, because we observed similar VT colonization between HIV positive and HIV negative children in our study population, it is unlikely that the addition of this data would significantly alter our results.

## Conclusions

Vaccine-type pneumococcal colonization continues to be common among children with and without pneumonia post-PCV10 introduction in Mozambique. The inclusion of children with pneumococcal clinical syndromes along with the use of molecular detection in culture negative NP specimens in future colonization studies could improve assessment of circulating disease-causing serotypes in children.

## Supporting information

S1 Table Pneumococcal carriage dataset. (XLS)
